# Proposed Dental Law

**Published:** 1898-04

**Authors:** 


### Editorial, Etc.

### PROPOSED DENTAL LAW.

**Note April 8, 1898. The Legislature has adjourned and the Dental Law of 1896 remains unchanged.**

The following are the proposed amendments to the dental law of 1896. Both have been referred to the Senate Committee on Sanitary Condition of the State. The first emanates from the Maryland State Dental Association and was read February 17, 1898; the amendments to Section 12 of Article 32 of the Code was introduced (March 4, 1898), by request of the attorney who defended the party fined $100 by the Criminal Court of Baltimore City. This judgment has been affirmed by the Supreme Bench of Baltimore City, and the Court of Appeals of Maryland.

A BILL. Entitled An Act to repeal Article 32, of the Code of General Laws of Maryland, title, "Dentistry," as re-enacted and amended by Chapter 378, of the General Assembly of Maryland, passed at the January session, 1896, and to re-enact said Article with amendments.

SECTION 1. Be it enacted by the General Assembly of Maryland, That Article 32, of the Code of Public General Laws, title "Dentistry," as re-enacted and amended by Chapter 378, of the Acts of the General Assembly of Maryland, passed at the January session, 1896, be, and the same is hereby repealed, and re-enacted, so as to read as follows:

1. It shall be unlawful for any person to practice or to attempt to practice dentistry in this State, within the meaning of the penalties of this Act, unless such person shall previously have obtained a certificate, as hereinafter provided, and unless such person shall place and exhibit on the door or other front part of the building, wherein such person shall so practice or attempt to practice dentistry, a sign or signs, on which shall be the name of such person.

SEC. 2. There shall be a State Board of Dental Examiners, whose duty it shall be to carry out the purposes and to enforce the provisions of this Act. Said Board shall consist of six practicing dentists of recognized ability and honor, residents of the State of Maryland, who have held regular dental diplomas for at least five years The members of said Board, as at present constituted, shall hold office for the residue of the respective terms for which they may have been appointed. On the expiration of the term or terms for which any one or more of the members of said Board may have been appointed, and on the expiration of the term or terms for which any one or more of the members of said Board, appointed in pursuance of the provisions of this Act, shall have been appointed, the Governor shall appoint the successor or successors of said member or members of said Board, from a list of dentists duly qualified, as hereinbefore prescribed, of double the number of vacancies to be filled, proposed and submitted to him by the Maryland State Dental Association, and chosen by a majority vote of the members of said Association present at a meeting of said Association, called for that purpose, of which meeting at least two weeks' notice stating the time, place and purpose thereof, shall be given to the members of said Association, and mailed to the addresses of said members by the Secretary thereof. The term for which the members of said Board shall be appointed, as aforesaid, shall be six years, and until their successors shall have been appointed and qualified. In case of any vacancy occurring in said Board by reason of the death, incapacity or neglect or refusal to act of any member thereof, or in any other way, the Governor shall, from a list of dentists duly qualified, as hereinbefore prescribed, of double the number of vacancies to be filled, submitted and proposed to him by the Maryland State Dental Association, and chosen as hereinbefore provided, appoint a successor or successors of said member or members, who shall hold office for the remainder of the unexpired term or terms of said member or members. Any member of said Board who shall be absent from two successive regular meetings thereof shall cease to be a member thereof.

SEC. 3. Said Board shall choose one of its members President, and one Secretary thereof, whose duties shall be those usually appertaining to their respective offices. But the Secretary of said Board shall, in addition to said duties be the legal custodian of all the property, moneys, minutes, records, proceedings and the seal of said Board. Said Board shall hold regular meetings in the months of May and November in each year, and special meetings as it may deem necessary. A majority of said Board shall constitute a quorum, but a less number may adjourn from time to time. The proceedings thereof shall at all reasonable times be open to public inspection. Any examination paper, submitted by an applicant for registration as hereinafter provided, shall at all reasonable times be open to the inspection of the members of the Maryland State Dental Association and of the applicant. Said Board shall adopt and have a seal inscribed as follows: Maryland State Board of Dental Examiners, 1884. Said Board shall annually on or before the first day of December in each year, make to the Governor, a report of its proceeding in writing.

SEC. 4. Transcripts from the book of registration, hereinafter provided for, certified by the Secretary of said Board, and sealed with its seal, and all certificates issued in pursuance of the terms of this Act, shall be evidence in any court of this State.

SEC. 5. Any person of good moral character, twenty-one years of age, who has graduated at, and holds a diploma from any university or college, incorporated and authorized to grant diplomas in dental surgery, by and under the laws of the United States, or of any one of the United States, and who is desirous of practicing dentistry in this State, may make application in writing to said Board, to be examined by it, with reference to his or her qualifications to practice dentistry, as aforesaid; and upon passing an examination satisfactory to said Board, which examination shall be in writing so far as practicable, his or her name and residence or place of business shall be registered in a book kept for that purpose, and a certificate shall be issued to such person, which certificate shall be signed by the officers of said Board and shall bear its seal, and shall further state the name of the person to whom the same is issued, the name of the university or college at which such person has graduated, and from which he or she holds a diploma, the date thereof, and the date of the issuance of the certificate. At the discretion of said Board, any applicant otherwise qualified may be registered without being subjected to such examination; and any person who has graduated at and holds a diploma from any university or college, incorporated and authorized to grant diplomas in dental surgery, by, and under the laws of the United States, or of any one of the United States, and who has after examination (but not otherwise) been duly registered and licensed to practise dentistry by a Board of Dental Examiners, created and existing under the laws of the United States, or of any one of the United States, shall be registered by said Board without being subjected to such examination.

SEC. 6. A fee of ten dollars shall be paid to the Secretary of said Board at the time of his or her application by any applicant for examination or registration, or both, as often as he or she shall apply for the same, which money shall be expended by the Board in defraying its expenses.

SEC. 7. A temporary certificate for a limited period of time, to be specified therein, may be issued by the officers of said Board to any applicant who is the holder of a regular dental diploma, and who has been duly registered by a Board of Dental Examiners, created and existing under the laws of the United States, or of any one of the United States, or to any applicant who has graduated at, and who holds a diploma from any university or college incorporated under the laws of the State of Maryland, and authorized to grant diplomas in dental surgery. But no such certificate shall be issued for a longer time than until the next regular meeting of said Board. The fee for the issuance of said temporary certificate shall be five dollars, which shall be returned to said applicant in case he or she shall pass an examination satisfactory to said Board, at the next examination succeeeding the issuance of such temporary certificate.

SEC. 8. Every person shall be said to be practising dentistry within the meaning of the penalties of this Act who shall, either for pay in any form or gratuitously, perform any operation, or part of operation, of any kind pertaining to the human mouth, treat diseases of the human jaws or teeth, or correct malpositions thereof; or who shall advertise, or hold himself out to practise dentistry or dental surgery in any of its branches, by putting out a sign or in any other manner.

SEC. 9. Any person who shall violate any of the provisions of this Article, shall be deemed guilty of a misdemeanor, and shall, upon conviction thereof, be fined not less than fifty dollars, nor more than three hundred dollars, or be confined not more than six months in Jaii, or both, in the discretion of the Court.

SEC. 10. Nothing in this Article shall be so construed as to interfere with the rights and privileges of physicians and surgeons duly licensed to practice their profession in this State, or of persons holding certificates duly issued to them by the State Board of Dental Examiners of Maryland, prior to the passage of this Act; or of dental students operating under the immediate supervision of their instructors in dental infirmaries of dental schools duly incorporated under the Laws of the State of Maryland.

SEC. 11. And be it enacted, That nothing contained in this Act, shall prevent or be so construed as to in any way to hinder the prosecution, conviction or punishment of any person who may have offended against any of the provisions of Article 32, of the Code of Public General Laws, title "Dentistry," or against any of the provisions of any of the Acts of the General Assembly of Maryland, of which the same was a codification, or of said Chapter 378, of the Acts of the General Assembly of Maryland, passed at its January Session, 1896.

SEC. 12. And be it enacted, That this law shall take effect from the date of its passage.

A BILL. Entitled An Act to repeal and re act with amendments, Section 12, of Article 32, of the Code of Public General Laws of Maryland, title "Dentistry."

SECTION 1. Be it enacted by the General Assembly of Maryland, That Section 12 of Article 32, of the Code of Public General Laws of Maryland be, and the same is hereby repealed and re-enacted so as to read as follows:

SEC. 12. Nothing in this article shall be so construed as to interfere with the rights and privileges of resident physicians and surgeons or with persons holding certificates duly issued to them prior to the 4th day of April, 1896, by a dental board created under the laws of any one of the United States, nor with any person who was engaged in the practice of dentistry prior to the thirty-first (31st) day of March, 1884, and who began the practice of dentistry in this State on or before the 4th day of April, 1896, nor with dental students operating under the immediate supervision of their instructors in dental infirmaries or dental schools created under the laws of this State.

SEC. 2. And be it enacted, That this Act shall take effect from the date of its passage.

